# A Close Relationship Between Networks of Interstitial Cells of Cajal and Gastrointestinal Transit *In Vivo*

**DOI:** 10.3389/fphar.2020.587453

**Published:** 2020-11-20

**Authors:** Kazuhisa Kishi, Moe Kamizaki, Noriyuki Kaji, Satoshi Iino, Masatoshi Hori

**Affiliations:** ^1^ Department of Veterinary Pharmacology, Graduate School of Agriculture and Life Sciences, The University of Tokyo, Tokyo, Japan; ^2^ Department of Pharmacology, School of Veterinary Medicine, Azabu University, Kanagawa, Japan; ^3^ Division of Anatomy and Neuroscience, Department of Morphological and Physiological Sciences, Faculty of Medical Sciences, University of Fukui, Fukui, Japan

**Keywords:** c-Kit, gastrointestinal motility disorders, interstitial cells of Cajal, spontaneous contraction, *W*/*W*^*v*^ mice

## Abstract

The interstitial cells of Cajal associated with the myenteric plexus (ICC-MP) are located in the same area as the myenteric plexus. ICC-MP networks are linked to the generation of electrical pacemaker activity that causes spontaneous gastrointestinal (GI) contractions; however, its role in GI transit is not clear. The aim of this study was to comprehensively investigate the effect of ICC-MP disruption on GI transit *in vivo* using *W*/*W* ^*v*^ mice, partially ICC-deficient model mice. In this study, we measured GI transit using a ^13^C-octanoic acid breath test, an orally administered dye and a bead expulsion assay. ICC were detected by immunohistochemical staining for c-Kit, a specific marker for ICC. Interestingly, we found that gastric emptying in *W*/*W* ^*v*^ mice was normal. We also found that the ability of small intestinal and colonic transit was significantly reduced in *W*/*W* ^*v*^ mice. Immunohistochemical staining using whole-mount muscularis samples revealed that c-Kit-positive ICC-MP networks were formed in wild-type mice. In contrast, ICC-MP networks in *W*/*W* ^*v*^ mice were maintained only in the gastric antrum and were significantly reduced in the ileum and colon. No significant changes were observed in the nerve structures of the myenteric plexus in *W*/*W* ^*v*^ mice. These findings suggest that ICC-MP contribute to GI transit as a powerful driving function *in vivo*.

## Introduction

Gastrointestinal (GI) transit is a complex process that integrates contraction, relaxation, and transit (Sanders et al., 2012). These integrative processes are *in vivo* functions responsible for the digestion and absorption of food as well as normal GI motility functions that maintain health in humans and animals. It is well known that GI motility is controlled by coordination between smooth muscle cells (SMC) and neural networks (Rattan, 1981). Recently, in addition to these cell groups, it was determined that interstitial cells of Cajal (ICC) produce a complicated locomotor pattern of the GI tract that is also attracting attention from the clinical standpoint (Blair et al., 2014; Iino et al., 2006; Sanders et al., 2014a).

The interstitial cells of Cajal associated with the myenteric plexus (ICC-MP) form cellular networks. ICC-MP have a pacemaker function to generate the GI locomotor pattern; signaling through the tyrosine kinase receptor c-Kit is important for development and differentiation in ICC (Huizinga et al., 1995; Sanders et al., 2006). To date, many *in vitro* studies have been conducted using electrophysiological analysis techniques to understand the role of pacemaker function played by ICC-MP. Previous reports have demonstrated that ICC-MP generate spontaneous and rhythmic slow waves that provide pacemaker activity to SMC via gap junctions (Cousins et al., 2003; Hennig te al., 2010; Kito et al., 2003; Sanders et al., 2014a). In addition, several reports showed that when ACK2, a monoclonal antibody that inhibits the function of the c-Kit receptor, was administered to mice starting immediately after birth, spontaneous contraction of the small intestine was reduced with the disappearance of ICC-MP and slow waves (Sato et al., 1996; Torihashi et al., 1995). These findings suggest that the slow waves produced by ICC-MP raise the membrane potential of SMC above the threshold of the smooth muscle action potential, which results in spontaneous contraction of SMC and contributes to the spontaneous movement of the GI tract. The ICC-MP networks could be strongly associated with the ability of GI transit *in vivo*.

The c-Kit gene mutant (*W*/*W* ^*v*^) mouse contains a loss-of-function mutation in the c-Kit gene and has been used in many studies as a classic animal model with partial loss of ICC (Chabot et al., 1988; Nocka et al., 1989). The presence of ICC in *W*/*W* ^*v*^ mice has been analyzed in detail for each site of the GI tract. In these studies, depletion of ICC-MP abolished the presence of pacemaker potential at the GI site, and ICC at other sites did not perform pacemaker functions (Hennig et al., 2010; Sanders et al., 2014a). Nevertheless, there is no unified view of GI transit in *W*/*W* ^*v*^ mice *in vivo*. What remains unclear is the following: 1) how does GI transit from the stomach to the large intestine in *W*/*W* ^*v*^ mice differ from that of wild-type (WT) mice? 2) Does GI transit correlate with the presence of ICC-MP? Clarifying the relationship between GI transit and ICC-MP is a prerequisite for developing therapeutic strategies for GI motility disorders targeting ICC networks that have not been achieved to date.

The aim of this study was to definitively demonstrate the contribution of ICC-MP to GI transit *in vivo*. We investigated the ability of GI transit at various sites in *W*/*W* ^*v*^ mice using multiple *in vivo* methods to determine whether loss of ICC-MP affected GI transit. Here, we present new findings for understanding the role of ICC-MP *in vivo*, demonstrating a close relationship between ICC-MP networks and GI transit.

## Materials and Methods

### Animals and Ethics Statement

All animal care and experimental procedures complied with the Guide for Animal Use and Care published by the University of Tokyo. All experimental protocols were approved by the Institutional Review Board of the University of Tokyo (approval code P18-131). C57BL/6J WT mice and *W*/*W* ^*v*^ mice (6–8 weeks old, male) were used in this study. *W*/*W* ^*v*^ mice were obtained from Sankyo Labo Service Corporation (Tokyo, Japan). Mice were housed under controlled conditions (22 ± 2°C on a 12 h light/dark cycle).

### Evaluation of Gastric Emptying

Gastric emptying in mice was evaluated using the ^13^C-octanoic acid breath test according to previous studies (Creedon et al., 2013; Kishi et al., 2019). In preparation for this test, mice were fasted for 18 h. The test meal was 200 mg of a solid meal consisting of heated egg yolk and 0.2 μL ^13^C-octanoic acid (Cambridge Isotope Laboratories, Inc., MA, United States). After administration of the test meal to the mice, breath samples were collected using a blow pump device (Thermo Fisher Scientific Inc., Tokyo, Japan). The breath samples were accumulated at a flow rate of 70 ml/min for 1 min and sent to a breath collection bag (Otsuka Pharmaceutical Co., Ltd., Tokyo, Japan).

### Data Analysis for the ^13^C-Octanoic Acid Breath Test

The ^13^C-labeled CO_2_ (^13^CO_2_) concentration in the breath was quantified to evaluate the results of the breath test. The changes in ^13^CO_2_ (Δ^13^C, ‰) were calculated from the ^13^CO_2_/^12^CO_2_ ratio in the breath collection bag, using an infrared spectroscopic analyzer (Otsuka Electronics Co., Ltd., Osaka, Japan). The value of Δ^13^C was used to calculate the maximum concentration (*C*_max_; ‰), the time to reach maximum concentration (*T*_max_; min) and the area under the exhalation concentration-time curve (AUC_240min_; ‰·min). The half-life (*T*_1/2_; min) was calculated from the slope of the elimination phase in the Δ^13^C curve. These parameters for the evaluation of gastric emptying were calculated according to previous reports (Kishi et al., 2019; Matsumoto et al., 2008).

### Determination of Small Intestinal Transit

Mice were fasted for 18 h in preparation for measurement of small intestinal transit. This assay to evaluate small intestinal transit *in vivo* was conducted according to previous reports (Kaji et al., 2018). The mice were orally dosed with 100 μL of 0.5% (w/v) fluorescein isothiocyanate (FITC)-dextran in saline. One hour after the procedure, the GI tract was separated into the stomach (Sto), the small intestine, the cecum (Cec), and the colon. The small intestine was divided into 10 equally sized segments. Similarly, the colon was also divided into 3 equally sized segments. These tissues were respectively homogenized, and the supernatants were used as extracts containing FITC-dextran. The fluorescence intensity of the extract was measured under excitation of 485 nm and emission of 535 nm. Finally, the geometric center (GC) value, an index of small intestinal motility, was calculated according to the following formula.

GC = Σ (% of total fluorescent signal per segment × segment number)/100.

### Determination of Colonic Transit

A bead expulsion assay was performed to measure murine colonic transit *in vivo*. Briefly, glass beads (Cellpoint Scientific Inc., Gaithersburg, MD, USA) were inserted into the distal colon under isoflurane anesthesia. The propulsion distance of the bead was 3 cm, and the colonic transit time was defined as the time from the waking of the mice to the ejection of the bead. Awakening from anesthesia is defined as awakening from a supine position to a self-induced prone position.

### Whole-Mount Muscularis Preparation

Whole-mount muscularis samples were prepared for immunohistochemical analysis. The antrum, ileum and distal colon were used in this study. The GI muscularis was gently dissected under a stereomicroscope. The muscularis sheet peeled from the GI tissue was fixed to a silicone cover and permeabilized with ice-cold acetone for 10 min. Fixed whole-mount preparations were washed with Tris-buffered saline (TBS). The TBS-washed preparation was used for immunohistochemical analysis.

### Immunofluorescence

Immunohistochemical staining was performed to assess networks of ICC and neurons, as described in previous reports (Kishi et al., 2019). Fixed whole-mount preparations were incubated with 2% bovine serum albumin in TBS to reduce nonspecific binding. The preparations were incubated with rat anti-c-Kit antibody (1:500 diluted clone; ACK2, Panapharm Laboratories, Kumamoto, Japan) and rabbit anti-PGP9.5 antibody (1:500 diluted UltraClone Limited, Isle of Wight, UK). The immunoreactivity was detected using an Alexa Fluor 488 or 594 secondary antibody (Life Technologies, Gaithersburg, MD, USA). The fluorescently labeled samples were imaged with a laser-scanning confocal microscope (EZ-C1, Nikon, Tokyo, Japan).

### Statistics

The numerical data were expressed as the mean ± standard error of the mean (SEM). Statistical differences were analyzed by an unpaired Student’s t-test for comparison between two groups. A value of *p* < 0.05 was regarded as significantly different.

## Results

### *W*/*W* ^*v*^ Mice Had Normal Gastric Emptying

**FIGURE 1:**
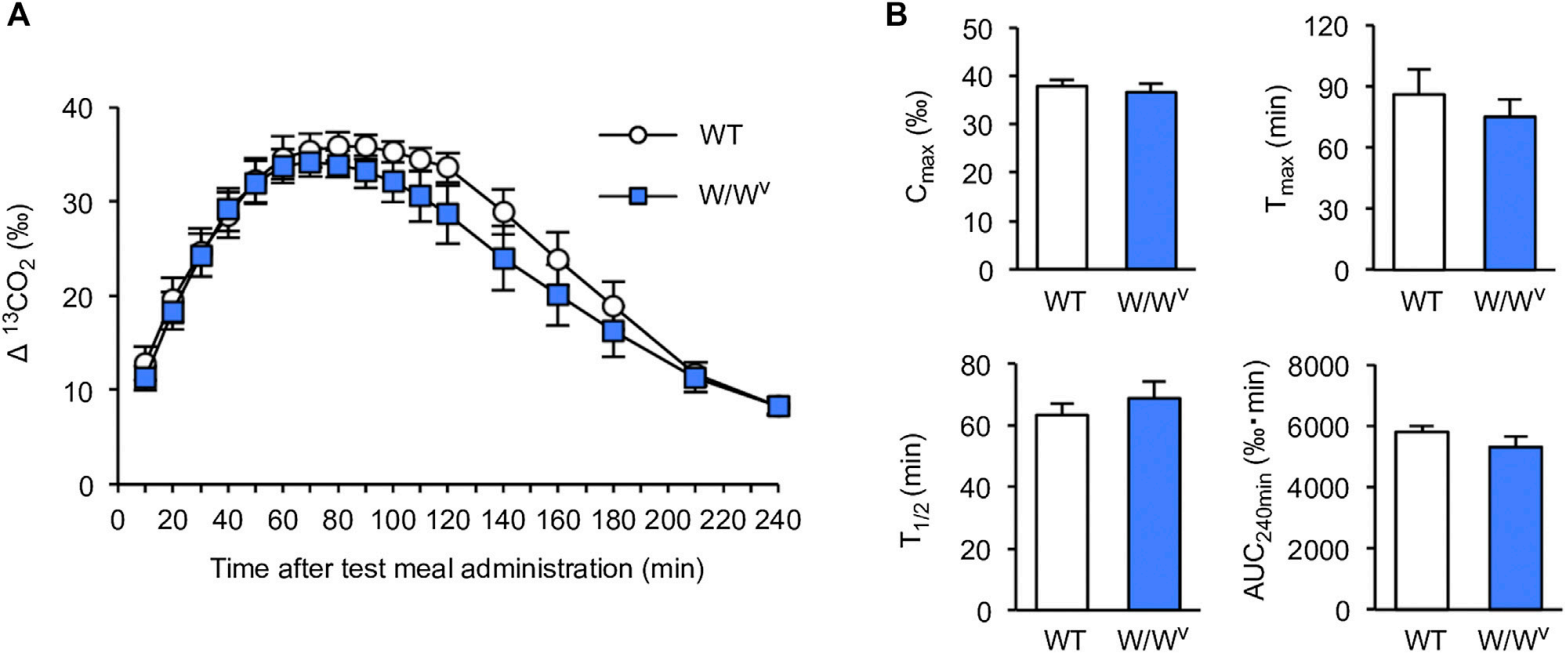
Normal gastric emptying in *W*/*W* ^*v*^ mice. **(A)** Time course of gastric emptying measured by the ^13^C-octanoic acid breath test in WT mice and *W*/*W* ^*v*^ mice. The measurement was continued for 4 h after the administration of the test meal. **(B)** Quantification of *C*_max_, *T*_max_, AUC_240min_, and *T*_1/2_ calculated from **(A)**. Each column shows the mean ± SEM (*n* = 5–6).

To investigate gastric-to-duodenal transit in *W*/*W* ^*v*^ mice, we measured gastric emptying using the ^13^C-octanoic acid breath test. In WT mice, the ^13^CO_2_ concentration increased slowly and peaked at approximately 90 min. The ^13^CO_2_ concentration after the peak began to decrease gradually and decreased to near the base value at 240 min. Interestingly, the ^13^CO_2_ excretion curve in *W*/*W* ^*v*^ mice showed changes in the ^13^CO_2_ concentration similar to those of WT mice (Figure 1A). Subsequently, to further evaluate the function of gastric emptying, we calculated *C*_max_, *T*_max_, AUC_240min_, and *T*_1/2_ as parameters of gastric emptying. When gastric emptying is delayed, *T*_max_ and *T*_1/2_ values increase, and *C*_max_ and AUC_240min_ values decrease. As a result of the measurement, there was no significant difference between WT mice and *W*/*W* ^*v*^ mice in all parameters (Figure 1B). These results suggest that the gastric emptying of *W*/*W* ^*v*^ mice is functionally normal.

### Small Intestinal Transit Was Decreased in *W*/*W* ^*v*^ Mice

**FIGURE 2:**
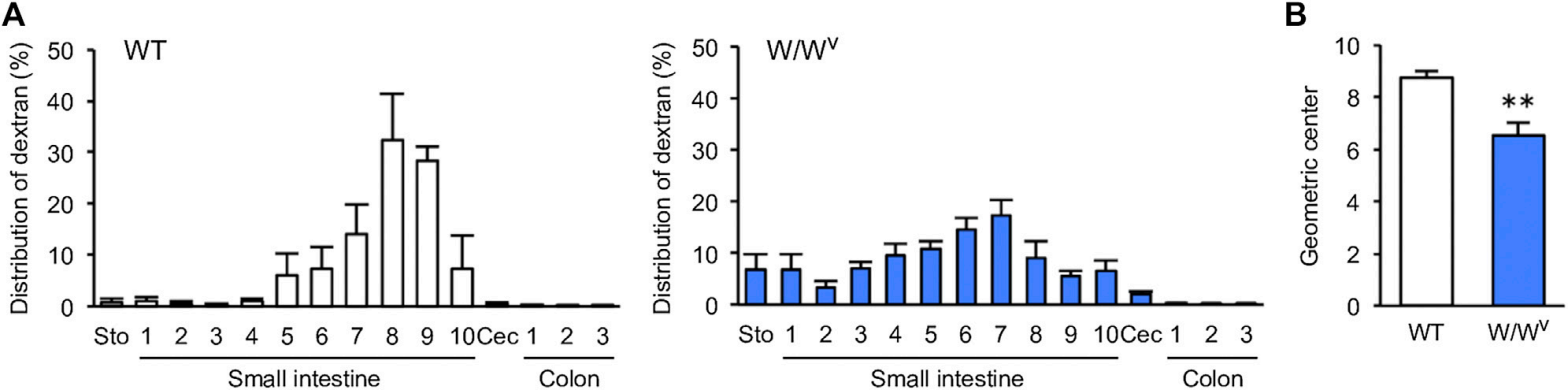
Decrease in small intestinal transit in *W*/*W* ^*v*^ mice. **(A)** Distribution of administered FITC-dextran in WT mice and *W*/*W* ^*v*^ mice. The small intestine was divided into 10 equal segments, and the colon was also divided into 3 equal segments. **(B)** Geometric center (GC) of each group calculated from **(A)**. Each column shows the mean ± SEM (*n* = 4–6). ^**^ *p* < 0.01; significantly different from WT mice.

Next, we evaluated the transit and distribution of orally administered FITC-dextran to investigate small intestinal transit in *W*/*W* ^*v*^ mice. In WT mice, FITC-dextran was distributed in the distal small intestine (Figure 2A, left). On the other hand, in *W*/*W* ^*v*^ mice, FITC-dextran was distributed in the middle parts of the small intestine (Figure 2A, right). In addition, the GC value calculated from the distribution of FITC-dextran was significantly lower in *W*/*W* ^*v*^ mice than in WT mice (Figure 2B). These results suggest that small intestine transit is reduced in *W*/*W* ^*v*^ mice.

### Colonic Transit Time Was Delayed in *W*/*W* ^*v*^ Mice

**FIGURE 3:**
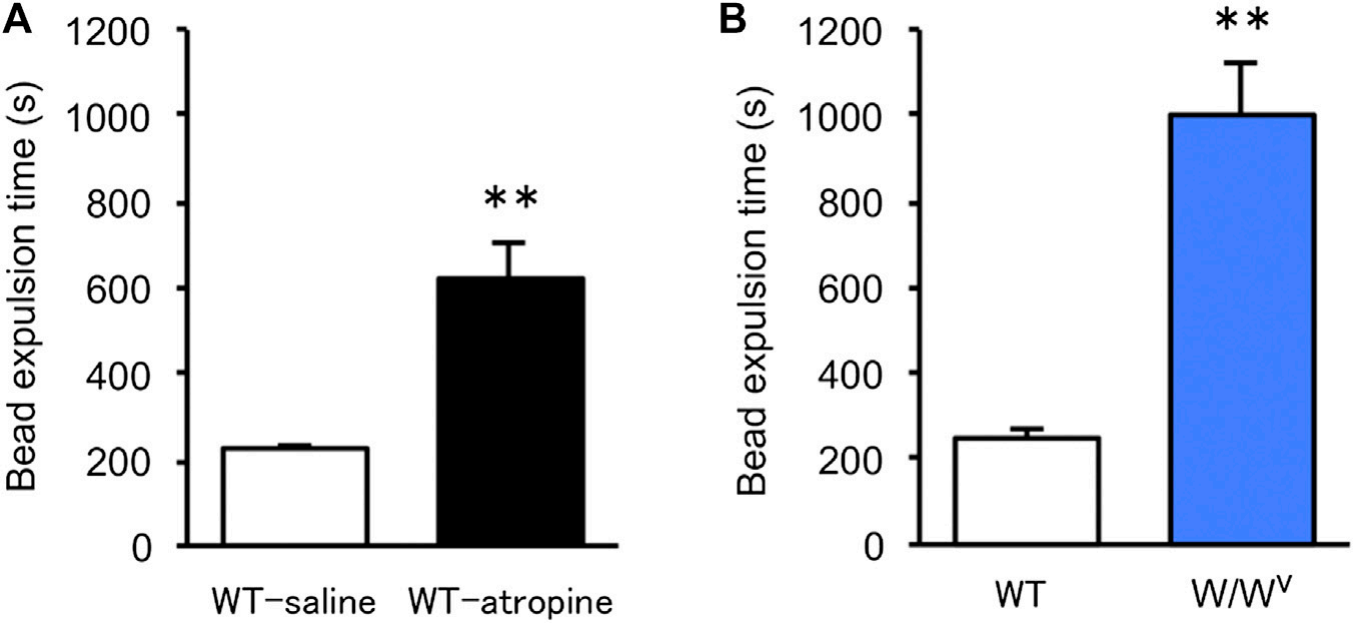
Delayed colonic transit time in *W*/*W* ^*v*^ mice. Colonic transit was assessed by the bead expulsion time. **(A)** Bead expulsion time in the saline group (WT mice administered saline) and atropine group (WT mice administered atropine). Each column shows the mean ± SEM (*n* = 6–8). **p < 0.01; significantly different from saline group. **(B)** Bead expulsion time in WT mice and *W*/*W* ^*v*^ mice. Each column shows the mean ± SEM (*n* = 7–8). **p < 0.01; significantly different from WT mice.

To evaluate colonic transit in *W*/*W* ^*v*^ mice, we measured colonic transit time by the bead expulsion assay. Atropine, an antidiarrheal drug, was used to confirm the delay in colonic transit time due to suppression of colonic motility. When atropine was subcutaneously administered at a concentration of 1 mg/kg, bead expulsion time was significantly delayed (Figure 3A). Based on these results, the colonic transit in *W*/*W* ^*v*^ mice was evaluated; the bead expulsion time was significantly delayed compared with that of WT mice (Figure 3B). The level of delayed colonic transit in *W*/*W* ^*v*^ mice was greater than the inhibitory effect of atropine. These results suggest that colonic transit is remarkably reduced in *W*/*W* ^*v*^ mice.

### ICC-MP Networks in *W*/*W* ^*v*^ Mice Were Normally Formed Only in the Antrum

**FIGURE 4:**
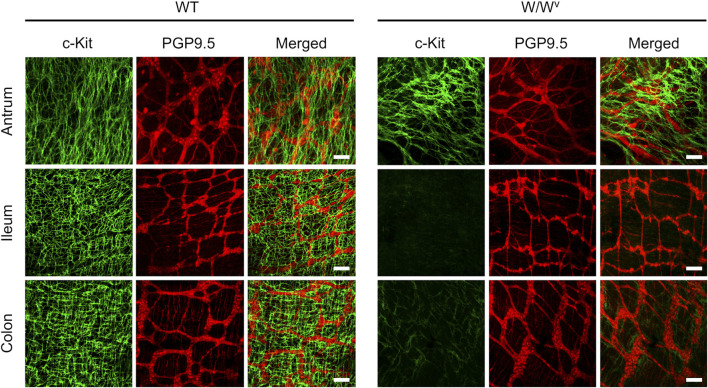
Changes in ICC of the myenteric plexus (ICC-MP) in *W*/*W* ^*v*^ mice. These images show representative results of double-immunostaining of c-Kit (ICC marker; green) and PGP9.5 (neuron marker; red) in whole-mount preparations of GI muscularis obtained from WT mice and *W*/*W* ^*v*^ mice. In the tissues of WT mice, ICC-MP expressing c-Kit (green) were located in the same area where is the myenteric plexus (red). In *W*/*W* ^*v*^ mice, ICC-MP were maintained only in the antrum and absent in the ileum and colon. Merged images are a combination of green and red color images. Scale bar, 100 μm.

Subsequently, to investigate changes in the ICC-MP in *W*/*W* ^*v*^ mice, we performed immunohistochemical staining using whole-mount samples of GI. In this study, c-Kit (green) and PGP 9.5 (red) were used as markers for ICC and neurons, respectively (Figure 4). In the tissues of WT mice, ICC-MP networks were associated with the myenteric plexus. ICC-MP were observed at all GI sites in the antrum, ileum and colon. In the tissues of *W*/*W* ^*v*^ mice, the distributions of ICC were different at each GI site, and ICC-MP were maintained only in the antrum. ICC-MP in the antrum of *W*/*W* ^*v*^ mice were not significantly reduced compared to the tissue of WT mice. On the other hand, ICC-MP in the ileum were remarkably reduced in *W*/*W* ^*v*^ mice. Similarly, in the distal colon, ICC-MP were significantly reduced in *W*/*W* ^*v*^ mice. In addition, there was no noticeable difference in the structure of neurons between the tissues of WT mice and those of *W*/*W* ^*v*^ mice, suggesting the integrity of the myenteric plexus in *W*/*W* ^*v*^ mice. These results suggest that in *W*/*W* ^*v*^ mice, the function of ICC-MP could be maintained only in the gastric antrum.

### ICC-Deep Muscular Plexus in Small Intestine Were Normally Observed in *W*/*W* ^*v*^ Mice

**FIGURE 5:**
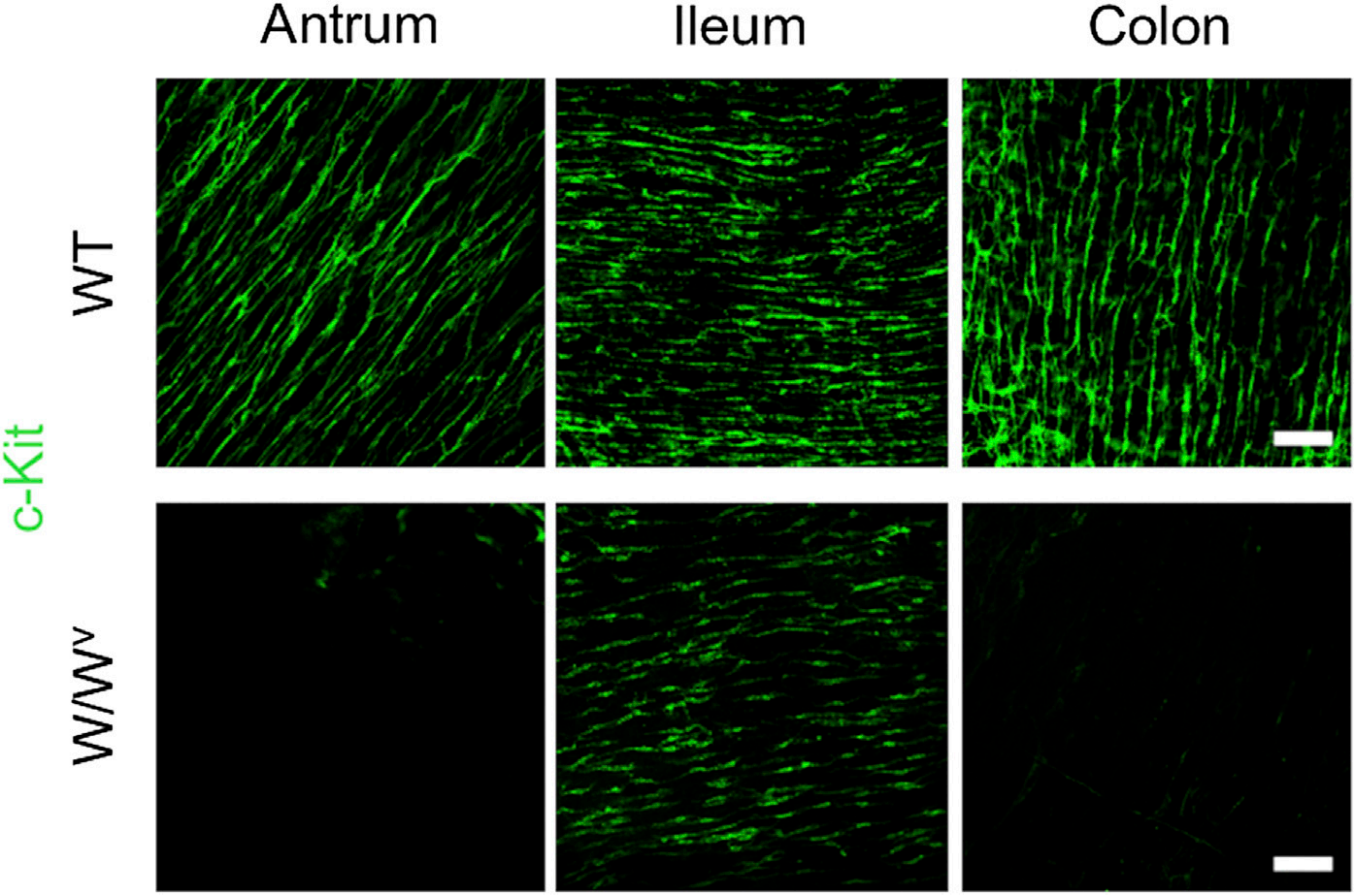
Changes in intramuscular ICC (ICC-IM; antrum and colon) or ICC associated with the deep muscular plexus in the circular muscle layer (ICC-DMP; small intestine) in *W*/*W* ^*v*^ mice. These images show representative results of immunostaining for c-Kit (ICC marker; green) in whole-mount preparations of GI muscularis obtained from WT mice and *W*/*W* ^*v*^ mice. Images in the antrum or colon show ICC-IM. Images in the ileum show ICC-DMP. ICC-IM or ICC-DMP were observed in the tissues of WT mice as c-Kit positive cells (green) in the antrum, ileum and colon. In the tissues of *W*/*W* ^*v*^ mice, ICC-DMP were observed in the ileum, and ICC-IM in the antrum and colon were absent. Scale bar, 100 μm.

Intramuscular ICC (ICC-IM; antrum and colon) or ICC associated with the deep muscular plexus in the circular muscle layer (ICC-DMP; small intestine) are known to be involved in the transmission of neural inputs from excitatory and inhibitory motor neurons (Hennig et al., 2010; Iino et al., 2009; Sanders et al., 2014a; Wang et al., 2005; Ward et al., 2006). To understand the relationship between GI transit and ICC-IM or ICC-DMP, we investigated changes in ICC-IM or ICC-DMP by immunohistochemical staining (Figure 5). ICC-IM or ICC-DMP were observed in the tissues of WT mice as c-Kit positive cells in the antrum, ileum and colon. On the other hand, in the tissues of *W*/*W* ^*v*^ mice, ICC-DMP were observed in the ileum. These results suggest that in *W*/*W* ^*v*^ mice, the transmission of neural input from neurons via ICC-DMP could be maintained only in the ileum.

## Discussion

Research on ICC has been the subject of GI research in recent years; however, the functional importance of ICC-MP that contribute to GI transit *in vivo* is not well understood. In this study, we demonstrated that GI transit *in vivo* is strongly associated with changes in ICC-MP. Interestingly, the function of gastric emptying in *W*/*W* ^*v*^ mice was normal. On the other hand, small intestinal and large intestinal transit were significantly delayed in *W*/*W* ^*v*^ mice. Furthermore, consistent with these results, we found that ICC-MP networks in *W*/*W* ^*v*^ mice were maintained only in the antrum. These observations suggest that ICC-MP networks contribute significantly to GI transit *in vivo*. The advantage of this study is that changes in GI transit were more closely investigated at each site of the GI tract by using multiple *in vivo* methods. ICC-MP networks could be effective targets in treatment strategies for GI motility disorders.

Many *in vitro* experiments using *W*/*W* ^*v*^ mice have shown a close relationship between ICC-derived slow waves and spontaneous contraction of the GI tract (Hennig et al., 2010); however, it is difficult to predict how abnormalities in ICC affect GI transit. The main reason for this is that GI transit is tightly controlled by the activation of multiple regulatory mechanisms, including the central and enteric nervous systems. This concern could contribute to the lack of a unified view of GI transit *in vivo*. In this study, we quantitatively analyzed GI transit in *W*/*W* ^*v*^ mice using multiple *in vivo* methods. The ^13^C-octanoic acid breath test revealed that gastric emptying of *W*/*W* ^*v*^ mice was normal. Previous reports have shown that electrophysiological measurements indicated that slow wave development in the stomach of *W*/*W* ^*v*^ mice is similar to that of WT mice (Hou et al., 2005; Ordög et al., 2002); this correlates with our data. These findings suggest that slow waves are rhythmically generated in the stomach of *W*/*W* ^*v*^ mice and that normal gastric emptying could be maintained *in vivo*. This study also showed that small and large intestinal transit in *W*/*W* ^*v*^ mice were significantly delayed compared to that of WT mice. Previously, strong evidence has been provided that there is impaired peristalsis in the small intestine of *W*/*W* ^*v*^ mice. Several studies reported that in *W*/*W* ^*v*^ mice, the slow contraction pattern observed in the normal small intestine was abolished by the disappearance of slow waves (Der-Silaphet et al., 1998; Hennig et al., 2010; Hou et al., 2005). This phenomenon was reproducible by continuous administration of ACK2 or imatinib, antibodies that inhibit the function of c-Kit (Beckett et al., 2007; Sato et al., 1996; Torihashi et al., 1999). In addition, a previous report revealed that in the large intestine, abnormalities in c-Kit signaling also caused decreases in the frequency of slow waves and irregular muscle contraction (Albertí et al., 2007). These lines of evidence strongly support the hypothesis that reduced pacemaker activity slows GI transit *in vivo* in the small and large intestines of *W*/*W* ^*v*^ mice. Taken together, these results suggest that GI transit in *W*/*W* ^*v*^ mice is characterized by pacemaker activity at each GI site. To the best of our knowledge, this study was the first *in vivo* study to comprehensively and completely characterize GI transit in *W*/*W* ^*v*^ mice. GI transit could be significantly affected by ICC-derived pacemaker activity.

ICC are classified into several subtypes based on their distribution within the gut muscle coat. ICC-MP are located in the same area where is the myenteric plexus and play the role of pacemaker in the stomach, small intestine, and large intestine (Sanders et al., 2006; Sanders et al., 2014a). This study revealed that ICC-MP networks formed normally only in the antrum in *W*/*W* ^*v*^ mice. Normal gastric emptying observed in *W*/*W* ^*v*^ mice could be maintained by rhythmic pacemaker activity by ICC-MP. In contrast to findings regarding the stomach, several studies have reported a decrease in slow waves and irregular contractile motility in the small and large intestines of ICC-deficient animal models (Albertí et al., 2007; Der-Silaphet et al., 1998; Hou et al., 2005). Consistent with this evidence, ICC-MP were significantly reduced in the ileum and colon of *W*/*W* ^*v*^ mice. These data support the findings of previous reports on the distribution of ICC and pacemaker function in *W*/*W* ^*v*^ mice (Okishio et al., 2005; Ward et al., 1994). These findings suggest that a reduction in ICC-MP is an important factor in delaying small and large intestinal transit *in vivo*. In this study, no significant changes were observed in the neural structure of the myenteric plexus in *W*/*W* ^*v*^ mice. A previous report showed that the neural function of the myenteric plexus in *W*/*W* ^*v*^ mice was normal (Huizinga et al., 1998). Therefore, changes in GI transit in *W*/*W* ^*v*^ mice are likely to be the result of being affected by ICC-MP, not changes in neural function.

ICC-IM and ICC-DMP are considered to have a function of transmitting input information from neurons to SMC (Hennig et al., 2010; Sanders et al., 2014a). In addition, we also showed that ICC-IM in *W*/*W* ^*v*^ mice were significantly reduced in the antrum and colon, and ICC-DMP were completely maintained in the ileum. Considering that there was no direct relationship between GI transit and the distribution of ICC-DMP and ICC-IM, ICC-DMP and ICC-IM seem to have a small effect on GI transit; however, ICC-DMP and ICC-IM at least play a mediating role between neurons and SMC and are known to have an additional effect on GI motility. ICC-MP generate omnipresent slow waves and ICC-DMP independently generate stimulus-dependent slow waves in the small intestine (Zhu et al., 2016). Therefore, the slow wave in *W*/*W* ^*v*^ mice is most likely coming from the ICC-DMP. A previous study reported that ICC-MP is predominant and may involve the slow waves generated by ICC-DMP (Jiménez et al., 2005). The present study showed that the small intestinal transit was delayed in *W*/*W* ^*v*^ mice, and it indicated that slow wave generating ICC-MP could be more important for transit. On the other hands, despite the loss of ICC-MP in the small intestine of *W*/*W* ^*v*^ mice, a moderate transit in small intestine was observed in the present study. One method of propulsion, the migrating motor complex (MMC), is directed by the enteric nervous system (Spencer et al., 2003), and the minute rhythm, a powerful propulsive motor pattern that occurs in the feeding state, likely involves the neural induction of slow wave like activity in the ICC-DMP (Wang et al., 2005). Therefore, the propulsive forces observed in *W*/*W* ^*v*^ mice could be supported by MMC and minute rhythm. ICC-IM also generates pacemaker waves and that the signal reaching the circular layer is enhanced by ICC-IM (Dickens et al., 2001). Therefore, ICC-MP and ICC-IM are important for the generation of complete slow waves. In the present study, we showed that *W*/*W* ^*v*^ mice have normal gastric emptying despite the lack of ICC-IM in the antrum. This suggests that the first component of ICC-MP-derived slow waves could be important for propulsion. The loss of both ICC-MP and ICC-IM could cause severe delays in GI transit. In fact, we identified a marked loss of both ICC subtypes and a significant delay in colonic transit in *W*/*W* ^*v*^ mice. At this stage, there is no conclusive evidence supporting the role of ICC-IM in GI transit. Additionally, ICC associated with the submuscular plexus (ICC-SMP) generate omnipresent slow-wave activity in the colon (Gomez-Pinillo et al., 2009; Hwang et al., 2009) and are associated with prominent motor patterns. This pacemaking was normal in the *W*/*W* ^*v*^ colon. Consistent with this view, previous reports have shown that Anoctamin1 (ANO1), the calcium-activated chloride channel, positive cells are maintained in ICC-SMP, but decreased in ICC-MP and ICC-IM whether they are c-Kit or Ano1-positive in *W*/*W* ^*v*^ mouse colon (Wang et al., 2014). Nevertheless, colonic transit in *W*/*W* ^*v*^ mice was markedly delayed in the present study; the extent to which ICC-SMP contribute to colonic transit could not be significant. Further studies will be needed to understand the role of ICC-IM and ICC-SMP *in vivo* in the future. Taken together, these findings suggest that GI transit is strongly dependent on the presence of ICC-MP networks.

Disorders of GI transit are induced by abnormal GI motility caused by various diseases and drugs that cause clinical symptoms such as vomiting, abdominal pain, anorexia, constipation, and diarrhea (Mearin et al., 2016; Stanghellini et al., 2016). GI symptoms are routine health problems in human clinical practice and in veterinary clinical practice including dogs, cats and horses. Recently, studies in animal models of diabetes and inflammation have provided additional information on the relationship between changes in ICC-MP and GI motility disorders. Previous studies suggested that loss of ICC-MP is associated with many GI motility disorders such as diabetic gastroparesis, ulcerative colitis, and ileus (Choi et al., 2008; Kaji et al., 2018; Lu et al., 2019). In addition, recent reports have shown that increased ICC-MP are associated with rapid gastric emptying, which is often observed in diabetes (Hayashi et al., 2017; Kishi et al., 2019). The present study conclusively showed that GI transit was strongly influenced by ICC-MP networks *in vivo*. Taken together, these findings suggest that changes in ICC-MP in various diseases are important determinants affecting GI transit. Future studies, including the validation of pathologies in various animal models, will help identify pathogenic factors that alter ICC-MP and their applications. On the other hand, this study has some limitations. The *W*/*W* ^*v*^ mice used in this study are congenital animal models with ICC deficiency, and other cells could compensate for the function of GI motility during postnatal development. That is, it is necessary to consider that the function of the cell group responsible for GI motility may be different from that of healthy mice. In addition, gastric emptying was determined by experiments focusing on the antrum; however, gastric adaptive relaxation in the fundus also affects gastric emptying (Kelly, 1980). ICC-IM in the fundus of *W*/*W* ^*v*^ mice are reduced (Sanders et al., 2014b), and loss of ICC-IM in the fundus leads to lack of normal adaptive relaxation, which could cause rapid gastric emptying (Ordög et al., 2000). To evaluate gastric emptying more strictly, additional experiments focusing on the motility of the whole stomach will be necessary. Although challenges remain, ICC-MP are closely linked to GI transit *in vivo* and could be a useful target for coordinating GI motility.

In conclusion, this study demonstrated that ICC-MP contribute to GI transit as a powerful driving function *in vivo*. Substantial loss of ICC-MP is a common feature of delays in GI transit caused by spontaneous peristaltic dysfunction. Disorders of GI transit caused by various diseases and drugs could be strongly associated with disruptions of ICC-MP networks. Our data provide new insights into the role of ICC-MP in GI transit *in vivo*.

## Data Availability Statement

The raw data supporting the conclusions of this article will be made available by the authors, without undue reservation.

## Ethics Statement

All animal care and experimental procedures complied with the Guide for Animal Use and Care published by the University of Tokyo. All experimental protocols were approved by the Institutional Review Board of the University of Tokyo (approval code P18-131).

## Author Contributions

KK and MH designed the study and wrote the paper. KK and MK performed experiments and analyzed the data. NK and SI provided many techniques and insights in this experiment. All of the authors reviewed and revised the manuscript. KK and MK these authors have contributed equally to this work.

## FUNDING

This work was supported by a Grant-in-Aid for JSPS Fellows (20J11240 to KK) and Scientific Research from The Ministry of Education, Culture, Sports, Science and Technology (24248050 to MH).

## Conflict of Interest

The authors declare that the research was conducted in the absence of any commercial or financial relationships that could be construed as a potential conflict of interest.
